# Fibroblast CEBPD/SDF4 axis in response to chemotherapy-induced angiogenesis through CXCR4

**DOI:** 10.1038/s41420-021-00478-0

**Published:** 2021-05-06

**Authors:** Jhih-Ying Chi, Yu-Wei Hsiao, Hai-Ling Liu, Xin-Juan Fan, Xiang-Bo Wan, Tsung-Lin Liu, Sheng-Jou Hung, Yi-Ting Chen, Hsin-Yin Liang, Ju-Ming Wang

**Affiliations:** 1 Department of Biotechnology and Bioindustry Sciences, College of Bioscience and Biotechnology, National Cheng Kung University, Tainan, 701 Taiwan; 2 Department of Pathology, The Sixth Affiliated Hospital of Sun Yat-sen University, Guangzhou, 510655 China; 3 Department of Radiation Oncology, The Sixth Affiliated Hospital of Sun Yat-sen University, Guangzhou, 510655 China; 4 International Research Center for Wound Repair and Regeneration, National Cheng Kung University, Tainan, Taiwan; 5 Graduate Institute of Medical Sciences, College of Medicine, Taipei Medical University, Taipei, 110 Taiwan; 6 Graduate Institute of Medicine, College of Medicine, Kaohsiung Medical University, Kaohsiung, 807 Taiwan

**Keywords:** Cancer microenvironment, Tumour angiogenesis

## Abstract

Cancer-associated fibroblasts (CAFs) play an essential role in supporting cancer progression. However, the details and consequent effects in response to the communication between CAFs and angiogenesis remain largely uninvestigated, especially in anticancer drug treatments. We found that cisplatin and 5-fluorouracil could induce fibroblast differentiation toward myofibroblasts via CCAAT/enhancer-binding protein delta (CEBPD) and consequently promote proliferation, migration, and in vitro tube formation of vascular endothelial cells and angiogenesis in vivo. Stromal-cell-derived factor 4 (SDF4) is responsive to anticancer drugs via CEBPD activation in CAFs and contributes to create a permissive environment for tumor cell angiogenesis and promotion of distant metastasis. Importantly, we demonstrated that SDF4 interacts with CXCR4 to trigger VEGFD expression through the activation of the ERK1/2 and p38 pathways in endothelial cells. Taken together, our novel findings support that SDF4 can be a therapeutic target in inhibition of angiogenesis for chemotherapy drug-administrated cancer patients.

## Introduction

Lung cancer is the most common malignancy worldwide^1^, and nonsmall cell lung cancer (NSCLC) represents 85% of lung cancer cases. Impaired vascularity has been observed in NSCLC and other cancer types. Meanwhile, the number of microvessels in tumors has been linked to the close relationship between vascularization and poor clinical prognosis in NSCLC. In addition to resulting in regions of acidosis and hypoxia, immature angiogenesis can lead to increased metastasis and treatment resistance^2^. A desmoplastic response occurs in most NSCLC tumors^3,4^. The tumor microenvironment consists of non-cancer cells, including stromal cells, vascular endothelial cells, and immune cells, and the extracellular matrix. Increasing evidence indicates that stromal alterations in cancers, including NSCLC, are characterized by the differentiation of fibroblasts into myofibroblasts, which promote the deposition of endothelial cell medium (ECM) in tumors, and angiogenesis^5,6^.

Recent studies suggest that the tumor microenvironment is a prominent shelter for the population of surviving tumor cells following initial chemotherapy^7,8^. In other words, the microenvironment can facilitate the development of therapeutic resistance^9,10^. Fibroblasts/myofibroblasts constitute a major and common stromal component of the tumor microenvironment and promote the growth and invasion of cancer cells. Detected CAFs are predominantly myofibroblasts^11^. Myofibroblasts are differentiated and distinct from normal fibroblasts in their expression of alpha-smooth muscle actin (α-SMA). Recently, CAFs have been suggested to contribute to lung cancer development and local tissue invasion by promoting tumor growth and modulating drug responses^12,13^. In addition, studies investigating the role of the microenvironment in cancer progression and response to therapies, such as radiation and anticancer drugs, have been conducted^14–20^. For example, an increase in the number of microvessels has been reported to be associated with poor clinical prognosis in lung tumors following curative surgery^21^. Increasing evidence indicates that anticancer therapy alters the stromal compartment in tumors, including CAF markers (vimentin and αSMA), which are increased after chemotherapy treatment^14,19^. However, the mechanisms responsible for regulating myofibroblasts and CAFs and their interaction with endothelial cells, especially in response to anticancer drugs, have not been established.

CEBPD belongs to the CCAAT/enhancer-binding protein family and participates in tissue differentiation, metabolism, and immune response. Our findings and those of others have suggested that CEBPD plays a vital role in inflammatory disease^22–24^. Previously, CEBPD was thought to be a tumor suppressor due to its ability to promote growth arrest and apoptosis in certain cancer types, including leukemia, breast cancer, cervical cancer, and hepatocellular carcinoma^25^. Interestingly, CEBPD also plays a pro-tumorigenic role by promoting genome instability and anticancer drug resistance^26,27^, indicating that CEBPD serves as a tumor suppressor or tumor promoter depending on the cancer cell context. Moreover, in the tumor microenvironment, CEBPD plays a protumor role through inhibition of phagocytosis and enhancement of immunosuppression, stemness, metastasis, and invasion following certain stimuli, such as proinflammatory factors or anticancer drugs^28,29^. However, the details underlying the stromal CEBPD-related protumor effects among cancer cells and surrounding non-cancer cells, such as endothelial cells, remain largely uncharacterized in the context of chemotherapy.

Stromal-cell-derived factors (SDFs) refer to a group of proteins that are derived from stromal cells, including fibroblasts. Among the SDFs, SDF1 (C-X-C motif chemokine 12, CXCL12) is a known chemokine, and other SDFs, such as SDF2, SDF3, SDF4, and SDF5, are less well-defined. Interestingly, SDF4, also known as Cab45, is a member of the CREC protein family and contains six EF-hand motifs and calcium-binding motifs^30^. Similar to other CREC family members, several SDF4 isoform products are encoded by the SDF4 gene and localized in the cytosol, on the cell surface or secreted into the extracellular space. SDF4 was suggested to be regulated in Ca^2+^-dependent secretory cargo sorting pathways in the trans-Golgi network (TGN), exhibited increased expression in multiple types of cancer cells with higher proliferation and metastatic potential and was shown to promote cancer cell migration^31–33^. However, the detailed regulation of SDF4 and the SDF4-mediated effects on tumorigenesis, including its potent link with the metastatic potential of cancer cells and angiogenesis, remain an open question.

## Results

### CEBPD contributes to anticancer drug tolerance in fibroblasts

**Fig. 1:**
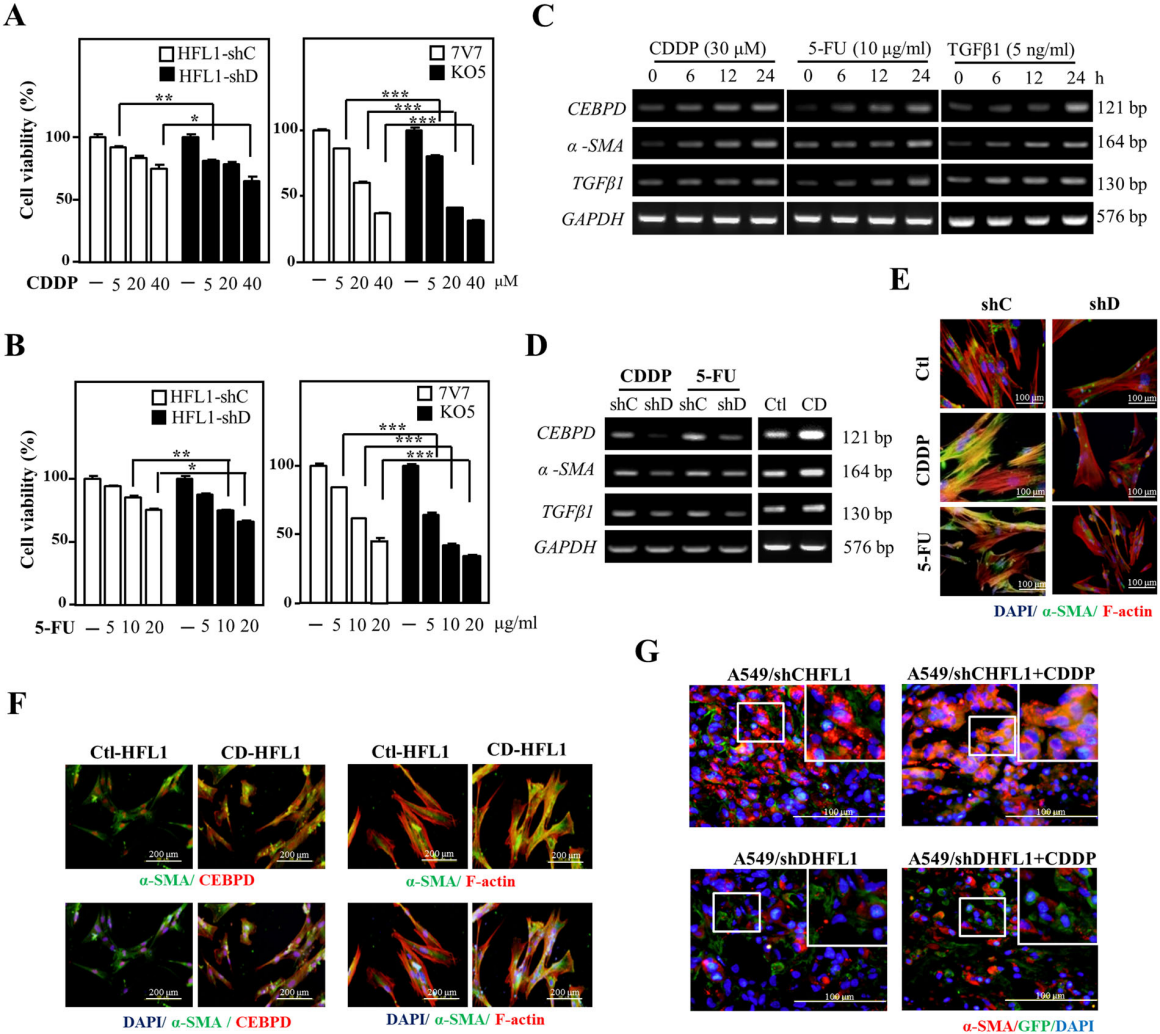
CDDP and 5-FU induce CEBPD and contribute to myofibroblast differentiation. **A**, **B** MTT assays were conducted to assess the cytotoxic effect of CDDP or 5-FU on HFL1, 7V7, and KO5 cells. HFL1 cells were infected with shβ-galactosidase (shC) or shCEBPD (shD) lentiviruses. Cells were seeded and their growth activity after treatment with CDDP or 5-FU for 24 h was compared. **C** An RT-PCR assay was performed with total RNA harvested from HFL1 cells treated with 30-μM CDDP, 10-μg/ml 5-FU, or 5-ng/ml TGFβ1. **D** HFL1 cells were infected with shC or shD lentiviruses and treated with or without CDDP or 5-FU for 6 h. For gain-of-function assays, total RNA was harvested from HFL1 cells infected with lentivirus bearing pAS3W-control (Ctl) or pAS3W-CEBPD expression vectors (CD). *CEBPD*, *α-SMA*, *TGFβ1*, and *GAPDH* transcripts were examined via RT-PCR. **E** HFL1 cells were infected with shC or shD lentiviruses and treated with or without CDDP or 5-FU for 6 h. **F** HFL1 cells were infected with lentivirus bearing Ctl or CD expression vectors. α-SMA expression and the formation of actin stress fibers were detected via immunofluorescence microscopy. **G** A549 cells mixed with HFL1 cells carrying a shβ-galactosidase knockdown vector with GFP (shC-GFP) or a CEBPD knockdown vector with GFP (shD-GFP) were inoculated subcutaneously into the dorsal rear flanks of NOD-SCID mice, and the mice were treated with or without CDDP (5 mg/kg). The mice with A549-xenografted tumors were sacrificed in the 12th week. Tumor tissues were stained for α-SMA (red) and GFP (green), and nuclei were stained with DAPI (blue). Three independent experiments were performed in triplicate. All data are expressed as the mean ± S.D. Differences among groups were analyzed with one-way ANOVA followed by the Tukey’s multiple comparison test. *p < 0.05, **p < 0.01, ***p < 0.001.

During anticancer drug treatment, stromal fibroblasts also face the challenge. However, the responses and consequent effects in this situation remain largely unknown. As mentioned above, stromal CEBPD enhances immunosuppression, stemness, metastasis, and invasion of cancer cells. However, the potential effect of stromal CEBPD on attenuating the cytotoxicity of anticancer drugs in fibroblasts and myofibroblasts and on communication with other non-cancer cells, such as endothelial cells, remain unknown. Following verification that CEBPD is responsive to cisplatin (CDDP) and 5-fluorouracil (5-FU) in fibroblasts (Fig. S1), we used the same experimental system combined with a loss-of-function assay to test the involvement of CEBPD in attenuating the cytotoxicity of CDDP and 5-FU in fibroblasts. The results showed that CEBPD knockdown HFL1 cells and *Cebpd*^−/−^ mouse embryo fibroblast (MEFs) (KO5 MEFs) were sensitized to CDDP and 5-FU compared with their individual experimental control HFL1 cells and *Cebpd*^+/+^ MEFs (7V7 MEFs) (Fig. 1A, B), suggesting that CEBPD contributes to anti-apoptosis of fibroblasts upon treatment of chemotherapeutic drugs.

### CEBPD contributes to CDDP- and 5-FU-promoted myofibroblast differentiation

A previous study showed that anticancer drugs increase the percentage of myofibroblasts^19^. Since fibroblast CEBPD is responsive to CDDP and 5-FU, we next tested whether activated CEBPD contributes to myofibroblast differentiation upon anticancer drug treatment. In addition to the anticancer drugs CDDP and 5-FU, we found that TGF-β could also induce the expression of CEBPD, α-SMA, and TGF-β in HFL1 fibroblasts (Fig. 1C). Meanwhile, exogenous induction of CEBPD upregulated *α-SMA* and *TGF-β1* transcripts (Fig. 1D), and the loss of CEBPD attenuated CDDP- and 5-FU-induced transcription of *α-SMA* and *TGF-β1* genes (Fig. 1D). These results indicate that TGF-β can exert positive feedback autoregulation following CDDP and 5-FU-induced CEBPD activation. Next, an immunofluorescence assay showed that CDDP and 5-FU treatments promoted the formation of stress fibers and enhanced CEBPD signals and α-SMA colocalization (Fig. 1E). We next assessed whether CEBPD could promote the formation of stress fibers and was associated with α-SMA abundance. The results show that exogenous CEBPD expression in fibroblasts promoted the formation of stress fibers (Fig. 1F) and enhanced α-SMA co-staining signals. We further assessed the in vivo effects of stromal CEBPD activation in cancer cells in response to CDDP treatment. First, A549 cells were cotransplanted with control knockdown or CEBPD knockdown HFL1 cells expressing green fluorescent protein (shC-HFL1 or shD-HFL1) into NOD-SCID mice. Compared with the xenografted tumors derived from A549 cells cotransplanted with shD-HFL1 cells, significant high α-SMA expression in fibroblasts was observed in xenografted tumors formed from A549 cells cotransplanted with shC-HFL1 cells upon CDDP treatment (Fig. 1G). These results suggest that CEBPD contributes to CDDP-induced α-SMA in HFL1 cells. In addition, it is well known that myofibroblasts show a mesenchymal phenotype. We next assessed whether CEBPD-expressing HFL1 cells showed mesenchymal features. Decreased *E-cadherin* (an epithelial marker) and increased *N-cadherin*, *Snail2*, and *Twist1* (mesenchymal markers) transcription (Fig. S1B) and enhanced motility (Fig. S1C) were observed in CEBPD-expressing HFL1 cells. The results suggest that anticancer drugs can activate fibroblasts toward myofibroblast differentiation and motility by activating CEBPD, at least in part.

### CEBPD-expressing fibroblasts promote angiogenesis and contribute to metastasis of lung cancer

**Fig. 2:**
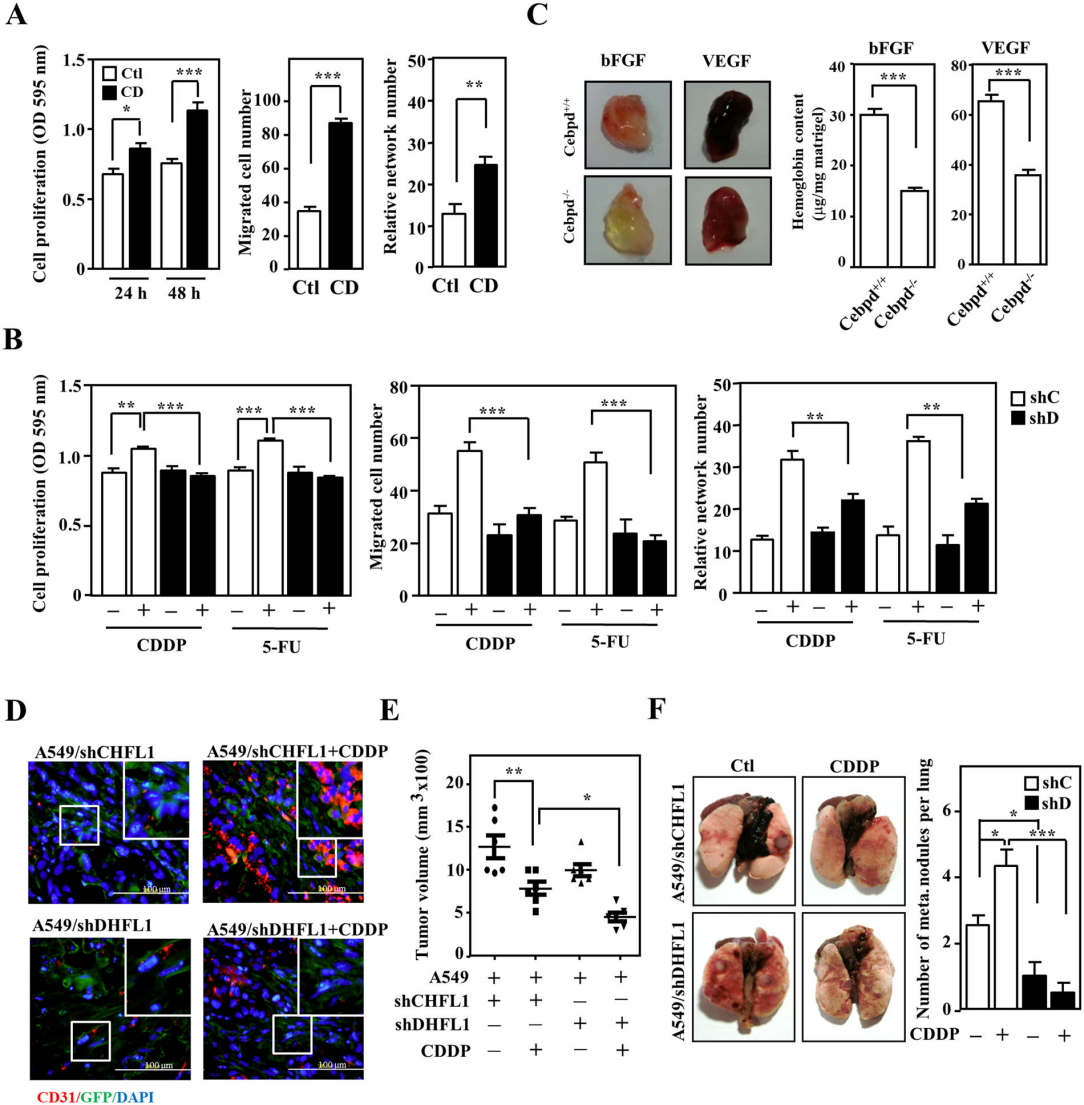
Activation of CEBPD in fibroblasts contributes to angiogenesis and metastasis of lung cancer. **A** HUVEC proliferation and migration were assessed using MTT and Boyden chamber assays, respectively. HUVECs were cultured in conditioned medium from HFL1 cells infected with lentivirus bearing pAS3W-control (Ctl) or pAS3W-CEBPD expression vectors (CD). The angiogenic effect was assessed by counting the number of intersections between branches of HUVECs grown in conditioned medium. The data are expressed as the mean ± S.D. Statistical analysis was performed using the Student’s *t* test: *p < 0.05, **p < 0.01, ***p < 0.001 versus the control group. **B** Assays to assess proliferation, migration, and in vitro tube formation were conducted. HUVECs were cultured with conditioned medium from HFL1 cells infected with shβ-galactosidase (shC) or shCEBPD (shD) lentiviruses as described in materials and methods. The data are expressed as the mean ± S.D. Statistical analysis was performed using one-way ANOVA followed by the Tukey’s multiple comparison test. **p < 0.01, ***p < 0.001. **C** *Cebpd*^+/+^ (WT) and *Cebpd*^–/–^ (KO) mice were subcutaneously inoculated with 800-ng/ml bFGF or 150-ng/ml VEGF Matrigel plugs. After 5 days, the mice were sacrificed, and the Matrigel plugs were removed to assess the newly formed blood vessels; hemoglobin levels were measured in the plugs using a Drakin’s reagent kit (*n* = 3 per group). The data are expressed as the mean ± S.D. Statistical analysis was performed using the Student’s *t* test: ***p < 0.001 versus the control group. **D** A549 cells mixed with shC-GFP or shD-GFP HFL1 cells were inoculated subcutaneously into the dorsal rear flanks of NOD-SCID mice, and the mice were treated with or without CDDP (5 mg/kg). The mice with A549-xenografted tumors were sacrificed in the 12th week. Tumor tissues were stained for CD31 (red) and GFP (green), and nuclei were stained with DAPI (blue). **E** Tumor volume was measured with external calipers and calculated using the standard formula: *V* = (*w* × *l*^2^) × 0.52, where *l* is the length and *w* is the width of the tumor (*n* = 6 per group). **F** The number of metastasis nodules from A549-xenografted tumors in the lungs was examined. Three independent experiments were performed in triplicate. The data are expressed as the mean ± S.D. Differences among groups were analyzed with one-way ANOVA followed by the Tukey’s multiple comparison test. *p < 0.05, **p < 0.01, ***p < 0.001.

Because they are neighbors in the tumor microenvironment, we speculated that endothelial cells do not just receive messages from cancer cells but also communicate with fibroblasts/myofibroblasts. Although previous studies indicated that metronomic chemotherapy decreases pro-angiogenic factors secretion in endothelial cells^34^, the details and regulation in endothelial cell–fibroblast communication to drug intervention is still unknown. We therefore tested whether CDDP- or 5-FU-treated fibroblasts could activate endothelial cell proliferation, migration, and in vitro tube formation. To investigate these issues, HUVECs were cultured with conditioned medium from HFL1 cells exogenously expressing CEBPD. The results showed that conditioned medium from fibroblasts expressing CEBPD promoted the proliferation, migration, and in vitro tube formation of HUVECs (Fig. 2A). Moreover, compared with conditioned medium from control knockdown HFL1 (shC-HFL1) cells, the proliferation, migration, and in vitro tube formation of HUVECs were attenuated by incubation with conditioned medium from CEBPD knockdown HFL1 (shD-HFL1) cells (Fig. 2B). VEGF and bFGF have been shown to synergistically regulate angiogenesis^35,36^. We recruited these two factors and conducted a matrigel plug assay to assess whether CEBPD in the microenvironment could enhance VEGF- and bFGF-involved angiogenesis. Compared with matrigel plugs in *Cebpd*^–/–^ mice, the matrix gel plugs in *Cebpd*^+/+^ mice exhibited a brighter red color when mixed with bFGF or VEGF angiogenetic factors (Fig. 2C), indicating that the loss of CEBPD impaired bFGF- and VEGF-induced angiogenic activities.

In addition, we further examined the effect of anticancer drug-activated fibroblast CEBPD induction on angiogenesis and metastasis of lung cancer cells. A549 cells were cotransplanted with shC-HFL1 or shD-HFL1 cells into NOD-SCID mice. After CDDP treatment, the expression of CD31 in endothelial cells of xenografted A549/shC-HFL1 tumors was significantly higher than that in xenografted A549/shD-HFL1 tumors (Fig. 2D). Moreover, although the size of both xenografted A549/shC-HFL1 and A549/shD-HFL1 tumors was reduced upon anticancer drug treatment (Fig. 2E), the metastasis/invasion of xenografted tumors was increased. Importantly, compared with xenografted A549/shD-HFL1 tumors, A549/shC-HFL1 tumors showed higher metastasis/invasion activity (Fig. 2F). Furthermore, we used a cancer cell allograft mouse model to confirm the contribution of mouse Cebpd in the microenvironment surrounding cancer cells. In comparison to tumors in *Cebpd* KO mice, the tumor growth and metastasis/invasion of luciferase expressing LLC1 cells (LLC1-Luc2)-bearing mice were significantly increased in *Cebpd* WT mice. The result suggests that the stromal CEBPD suppresses the growth and metastasis of LLC1 cells (Fig. S1D). Taken together, these results agree with our previous claim that stromal CEBPD plays a protumor role^28,29^ and provide new insight into how fibroblast CEBPD contributes to angiogenesis.

### CEBPD upregulates SDF4 in fibroblasts

To identify anticancer drug-induced fibroblast CEBPD responsive genes potentially involved in promotion of angiogenesis, a comprehensive transcriptome profiling analysis was performed using total RNA harvested from CDDP-treated shC-HFL1 and shD-HFL1 cells. We screened genes and compared them with 2,483 known secretory factors in the Secreted Protein Database (SPD). Eleven genes, including CCL20, with at least two-fold downregulated expression in CDDP-treated shD-HFL1 cells (Fig. S2A) were identified in response to CEBPD in HFL1 cells^22^. In addition to the most significant CEBPD responsive gene SDF4, several genes were selected to verify whether their transcription levels were indeed regulated by CEBPD. Q-PCR results show that *SDF4, CCL20, TGOLN2*, and *PSMB8* transcripts were positively regulated by CEBPD in CDDP-treated HFL1 cells (Fig. S2B).

**Fig. 3:**
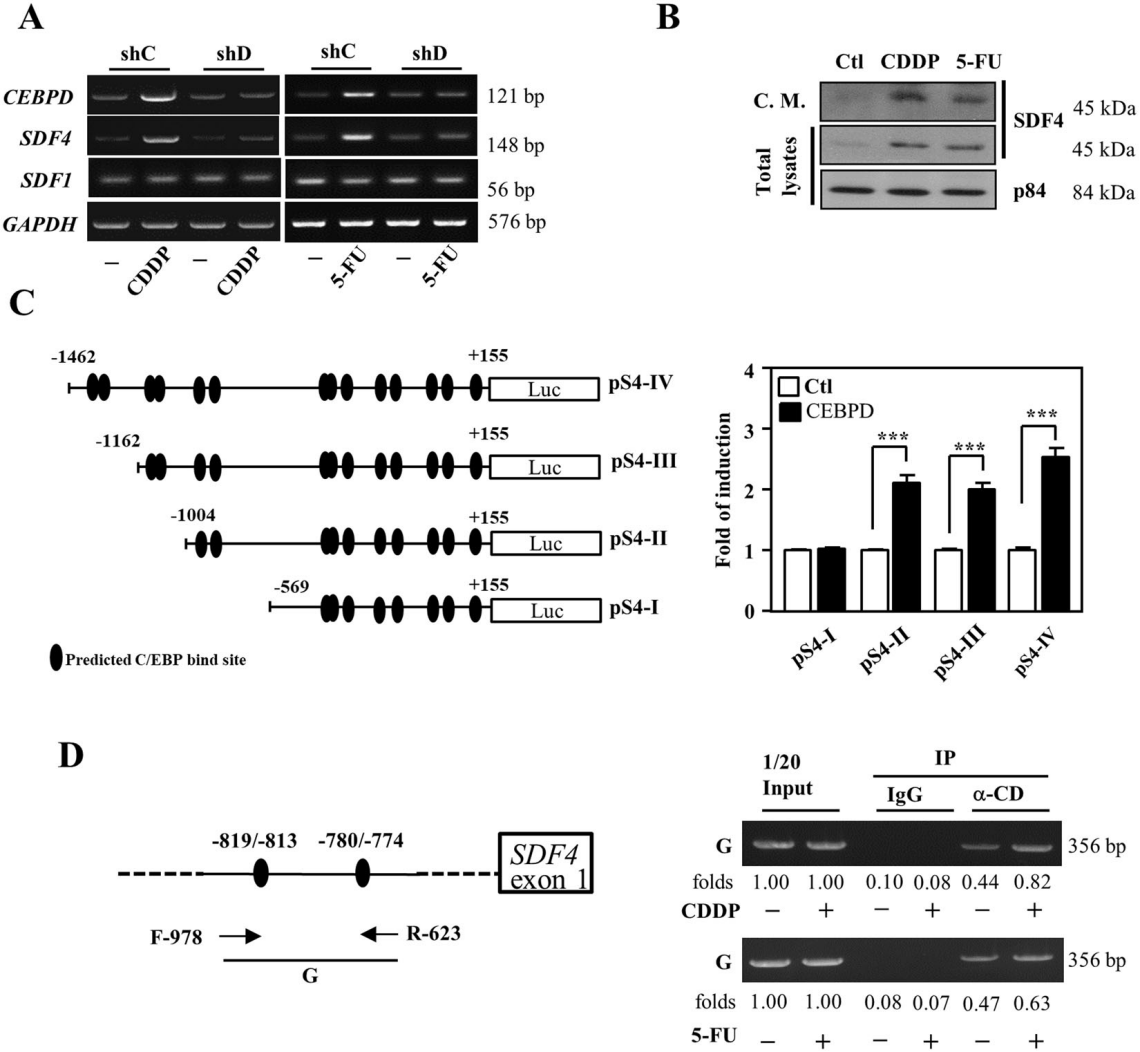
SDF4 is activated upon induction of CEBPD in lung HFL1 fibroblasts. **A** SDF4 expression was induced following CEBPD induction by CDDP or 5-FU treatment in HFL1 cells infected with shβ-galactosidase (shC) or shCEBPD (shD) lentiviruses and treated with or without CDDP or 5-FU for 6 h. RT-PCR assays were conducted to examine the *CEBPD*, *SDF4, SDF1*, and *GAPDH* transcript levels. **B** SDF4 expression was examined in conditioned medium (C.M.) or cell lysates from HFL1 cells after CDDP or 5-FU treatment for 6 h. p84 was used as an internal control. **C** CEBPD activates *SDF4* reporter activity. Representation of reporter constructs (left panel). A reporter assay was conducted to assess the activity of the *SDF4* reporter with or without CEBPD expression vector (right panel). **D** A ChIP assay was performed with the indicated antibodies. Three independent experiments were performed in triplicate. All data are expressed as the mean ± S.D. Differences between groups were analyzed with the unpaired two-tailed *t*-test. ***p < 0.001.

Previously, SDF1 has been suggested to play a pro-angiogenic role and contribute to cancer metastasis^37,38^. However, in contrast with SDF4, *SDF1* transcription was not responsive to CEBPD induction or CDDP treatment (Fig. 3A). Consistent with the increase in *SDF4* transcripts, increased SDF4 protein was also observed in CDDP- and 5-FU-treated HFL1 cells and their conditioned medium (Fig. 3B). Next, the CEBPD responsive region, −1004/-569 bp, on the promoter of *SDF4* gene was further identified using a serial deletion reporter assay (Fig. 3C). Finally, an in vivo DNA binding assay demonstrated that the binding of CEBPD onto the *SDF4* promoter was responsive to CDDP and 5-FU treatment in HFL1 cells (Fig. 3D). The results suggest that CEBPD can directly bind to the *SDF4* promoter and activate *SDF4* transcription in response to CDDP and 5-FU treatment in HFL1 cells.

### Fibroblasts-secreted SDF4 promotes angiogenesis of endothelial cells

**Fig. 4:**
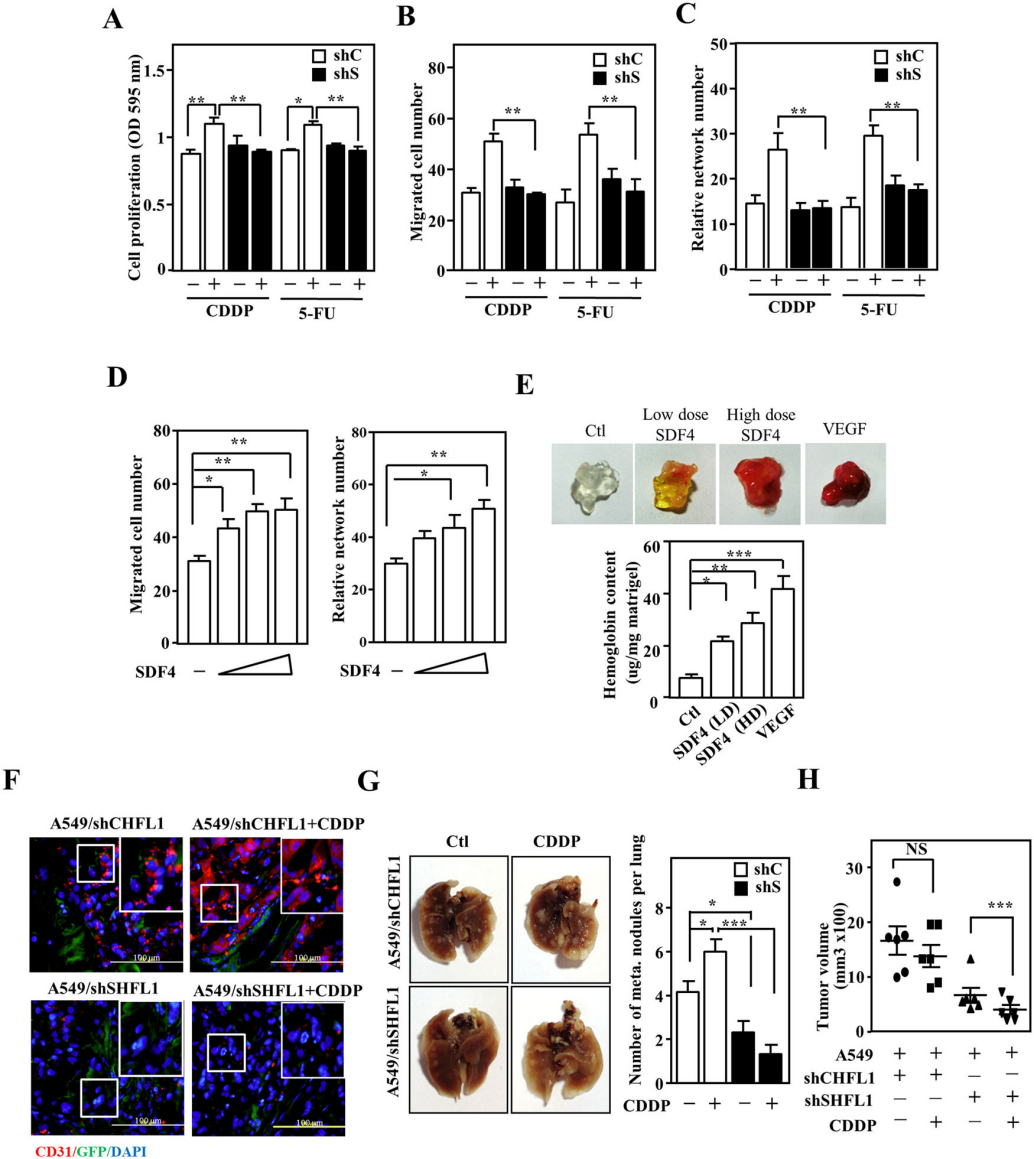
SDF4 participates in proliferation, migration, and angiogenesis of endothelial cells. **A** The proliferation of HUVECs cultured in conditioned medium from HFL1 cells infected with shβ-galactosidase (shC) or shSDF4 (shS) lentiviruses and treated with or without CDDP or 5-FU for 6 h, followed by 18-h recovery with fresh medium was assessed. **B** The migration of HUVECs was assessed by determining the number of HUVECs cultured in conditioned medium from HFL1 cells infected with shC and shS lentiviruses and treated with or without CDDP or 5-FU for 6 h, followed by 18-h recovery with fresh medium. **C** Angiogenesis was assessed by counting the intersection number between branches of HUVECs growing in conditioned medium from HFL1 cells infected with shC and shS lentiviruses and treated with or without CDDP or 5-FU for 6 h, followed by 18 h recovery with fresh medium. **D** Assays to assess migration and in vitro tube formation were conducted as described in materials and methods. HUVECs were treated with SDF4 at 0.25, 0.5, and 1 μg/ml. **E** *Cebpd*^+/+^ (WT) mice were subcutaneously inoculated with 0.5-μg/ml SDF4, 1-μg/ml SDF4, or 0.2-μg/ml VEGF Matrigel plugs. The experimental mice were sacrificed, and the Matrigel plugs were removed to assess the newly formed blood vessels; hemoglobin levels were measured in the plugs using a Drakin’s reagent kit (*n* = 3 per group). VEGF was used as a positive control. **F** A549 cells mixed with HFL1 cells carrying a shβ-galactosidase knockdown vector with GFP (shC-GFP) or a SDF4 knockdown vector with GFP (shS-GFP) were inoculated subcutaneously into the dorsal rear flanks of NOD-SCID mice, and the mice were treated with or without CDDP (5 mg/kg). The mice with A549-xenografted tumors were sacrificed in the 12th week. Tumor tissues were stained for CD31 (red) and GFP (green), and nuclei were stained with DAPI (blue). **G** The number of metastasis nodules from A549-xenografted tumors in the lungs was determined. **H** Tumor volume was measured with external calipers and calculated using the standard formula: *V* = (*w* × *l*^2^) × 0.52, where *l* is the length and *w* is the width of the tumor (*n* = 6 per group). Three independent experiments were performed in triplicate. All data are expressed as the mean ± S.D. Differences among groups were analyzed with one-way ANOVA followed by the Tukey’s multiple comparison test. *p < 0.05, **p < 0.01, ***p < 0.001.

To examine the important function of SDF4 in endothelial cells, we performed next-generation sequencing analysis of SDF4-treated HUVECs. Furthermore, to explore the effect of SDF4 on biological processes and signal transduction in endothelial cells, gene ontology process analysis was performed to elucidate the biological implications and unique genes that respond to SDF4. We found that genes relating to regulation of angiogenesis, vasculature development, cell division, cell motility, locomotion, cell migration, transmembrane receptor protein tyrosine kinase signaling pathway, and cell proliferation were significantly enriched in this gene set (Fig. S3A). By taking these results combined with those of CEBPD-expressing fibroblasts in pro-angiogenesis upon anticancer drug treatment (Fig. 2), we further tested whether SDF4 participates in anticancer drug-induced angiogenesis. Following culture with conditioned medium from CDDP- or 5-FU-stimulated HFL1 cells, enhanced proliferation, migration, and tube formation of HUVECs were observed. In contrast, these effects were attenuated when HUVECs were cultured in conditioned medium from HFL1 cells lacking SDF4 (Fig. 4A–C). Moreover, purified recombinant SDF4 showed a similar effect on promotion of proliferation, migration, and tube formation of HUVECs (Figs. S3B and 4D). In addition, we further assessed whether SDF4 could induce angiogenesis using a matrigel plug assay. The plugs mixed with increased concentrations of SDF4 in mice exhibited a brighter red color and significantly increased hemoglobin content (Fig. 4E). Furthermore, we examined whether anticancer drug-induced SDF4 in fibroblasts contributes to angiogenesis and metastasis of lung cancer cells. A549 cells were cotransplanted with shC-HFL1 cells or SDF4 knockdown HFL1 (shS-HFL1) into NOD-SCID mice. Following CDDP treatment, higher CD31 expression and number of metastatic A549 cells in the lungs were observed in xenografted tumors derived from A549 cells cotransplanted with shC-HFL1 cells (Fig. 4E, F). Meanwhile, the tumor size of A549/shC-HFL1 cells showed greater growth than A549 tumors cotransplanted with shS-HFL1 cells (Fig. 4G). Taken together, these finding suggest that anticancer drug can induce SDF4 expression in fibroblasts and consequently contribute to angiogenesis.

### SDF4 interacts with **C-X-C chemokine receptor type 4** (CXCR4) to induce VEGFD expression for angiogenesis by phosphorylating ERK and p38 pathway proteins in endothelial cells

**Fig. 5:**
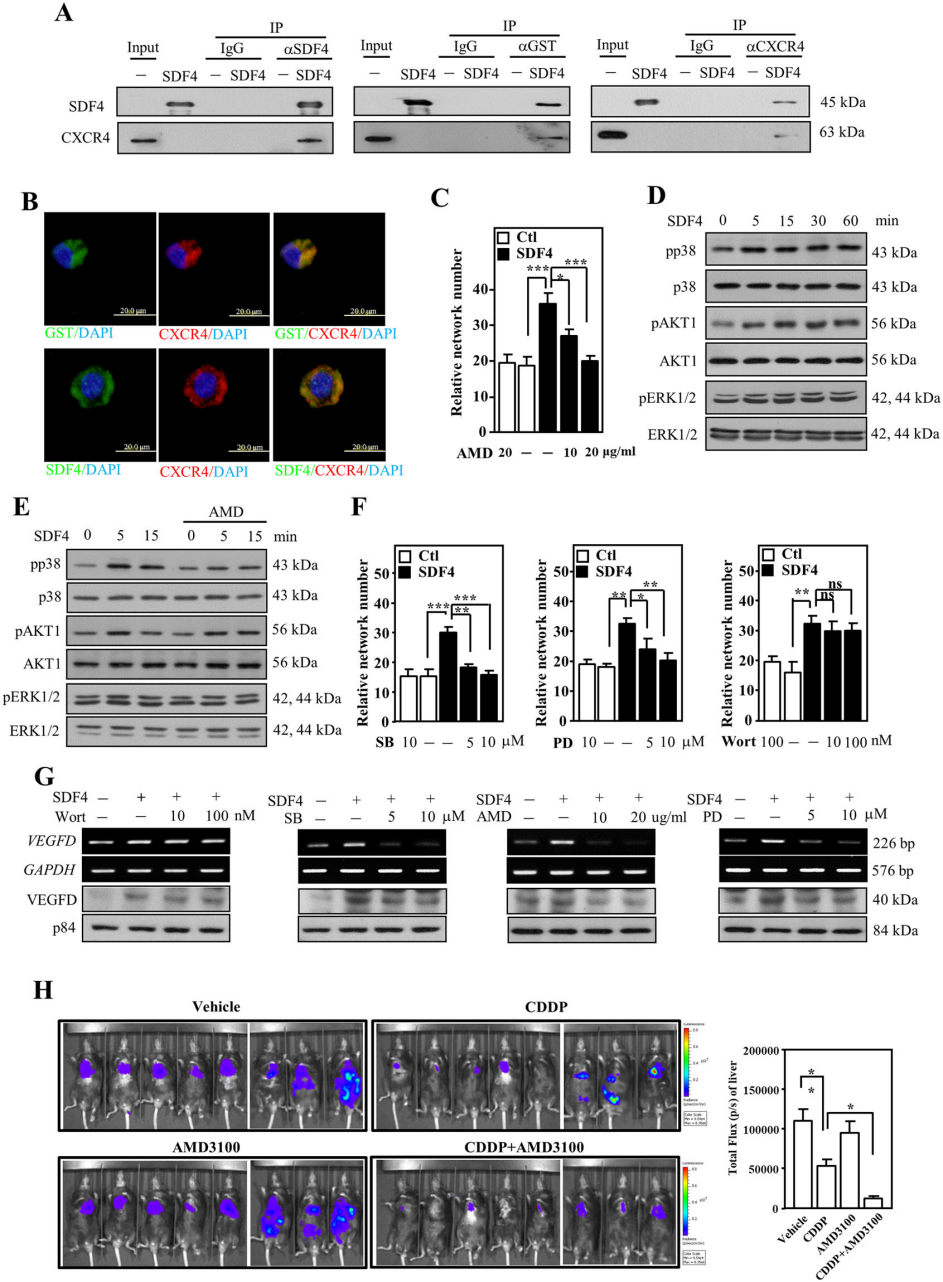
SDF4 interacts with CXCR4 receptor to induce VEGFD expression for angiogenesis by phosphorylating ERK and p38 pathway proteins in endothelial cells. **A** Cell membrane fractions were incubated with or without GST-SDF4, and then, a co-immunoprecipitation assay was performed with antibody against SDF4, GST or CXCR4. **B** An immunofluorescence assay was conducted to assess GST, SDF4, and CXCR4 signals. **C** Angiogenesis was assessed by counting the branched intersection number of SDF4-treated HUVECs with or without AMD3100 treatment as indicated. **D**, **E** The activity of AKT1, ERK1/2, and p38 in response to SDF4 (0.5 μg/ml) and with or without AMD3100 (20 μg/ml) treatment in the indicated time courses. **F** Angiogenesis was assessed by counting the branched intersection number of HUVECs treated with SDF4 and/or the indicated kinase inhibitors or SDF4 combined with pretreatment with increasing concentrations of wortmannin, PD98059 or SB203580. **G** VEGFD expression in response to various signaling inhibitors on SDF4-treated HUVECs. Three independent experiments were performed in triplicate. **H** LLC1-Luc2 cells were orthotopically inoculated into the lung of C57BL/6 mice. The experimental mice were treated with CDDP or AMD3100 as indication after inoculation with tumor cells. Representative in vivo bioluminescent images and total tumor flux of LLC1-Luc2-bearing mice in each group shown at 5th week. *n* = 8 per group. All data are expressed as the mean ± S.D. Differences among groups were analyzed with the one-way ANOVA followed by Tukey’s multiple comparison test. *p < 0.05, **p < 0.01, ***p < 0.001.

As mentioned above, SDF4 belongs to the SDF family. The SDF1/CXCR4 complex has been suggested to be involved in many cellular functions, including embryogenesis, immune surveillance, inflammation, tumor growth, and metastasis^39–41^. In our comprehensive transcriptome profiling analysis of CDDP-treated HFL1 cells, SDF1 had no response to CEBPD activation or CDDP treatment. We tested whether SDF4 could interact with CXCR4 and if CXCR4 could mediate SDF4-induced angiogenesis. To test the interaction between SDF4 and CXCR4, a co-immunoprecipitation assay was performed, and an interaction between SDF4 and CXCR4 was observed after incubation of recombinant SDF4 protein with the membrane fraction of HUVEC lysates (Fig. 5A). Next, purified recombinant SDF4 signals colocalized with CXCR4 signals on the HUVECs were observed in an immunofluorescence assay (Fig. 5B). Moreover, AMD3100, a CXCR4 antagonist, was recruited to assess whether inhibition of CXCR4 could attenuate the SDF4-induced pro-angiogenic effect. The results showed that AMD3100 dramatically inhibited the SDF4-induced tube formation of HUVECs (Figs. 5C and S4A). It implies that SDF4/CXCR4 complex plays a critical and specific role in chemotherapy-regulated angiogenesis, but not SDF1/CXCR4, and consequently contribute to metastasis of lung cancer. Several signaling pathways associated with AKT1, ERK1/2, and p38 activation have been reported to be involved in CXCR4-mediated responses. To clarify the involvement of signaling pathways in response to binding of SDF4 and CXCR4 and that consequently contribute to angiogenesis, AKT1, ERK1/2, and p38 activation was examined in HUVECs following SDF4 treatment. We found that SDF4 could activate AKT1, ERK1/2, and p38 signaling (Fig. 5D). Next, AMD3100 was recruited to assess the involvement of CXCR4 in SDF4-induced AKT1, ERK1/2, and p38 activation. Interestingly, AMD3100 specifically inhibited SDF4-induced ERK1/2 and p38 but not AKT1 activation (Fig. 5E), implying the existence of other SDF4 receptors on HUVECs. Moreover, in contrast with the non-response to wortmannin, a PI3K/AKT1 inhibitor, treatment with PD98059, an MEK1/ERK1/2 inhibitor, and SB203580, a p38 inhibitor, inhibited SDF4-induced tube formation of HUVECs (Figs. 5F and S4B), suggesting that ERK1/2 and p38 play functional roles in SDF4/CXCR4-induced pro-angiogenetic activity. As mentioned above, VEGFs and bFGF play a cooperative role in angiogenic activity. We further verified whether SDF4 could activate the transcription of *VEGF* and *bFGF* genes in HUVECs. We found that transcription of the *VEGF-D* gene but not the *VEGF-A*, *VEGF-B*, *VEGF-C*, and *bFGF* genes was specifically responsive to SDF4 in HUVECs (Fig. S4C). We also examined the involvement of CXCR4, ERK1/2, p38 in the SDF4-induced *VEGFD* transcription. The results showed that inhibition of CXCR4, ERK1/2, and p38 attenuated SDF4-induced *VEGFD* transcription in HUVECs (Fig. 5G). We further assessed the effects of CXCR4 by examining in vivo tumor growth and metastasis/invasion of LLC1 cells upon combining CDDP and AMD3100 treatment. The in vivo results show that CDDP monotherapy was efficacious and reduced primary tumor volume, but had only modest effect on liver metastasis. Specifically, combining CDDP and AMD3100 was completely inhibited liver metastasis, suggesting that chemotherapy-induced angiogenesis and metastasis could be block through potentially inhibiting SDF4/CXCR4 interaction (Fig. 5H).

### Correlations between SDF4 and cisplatin treatment, tumor angiogenesis, and patient outcomes

**Fig. 6:**
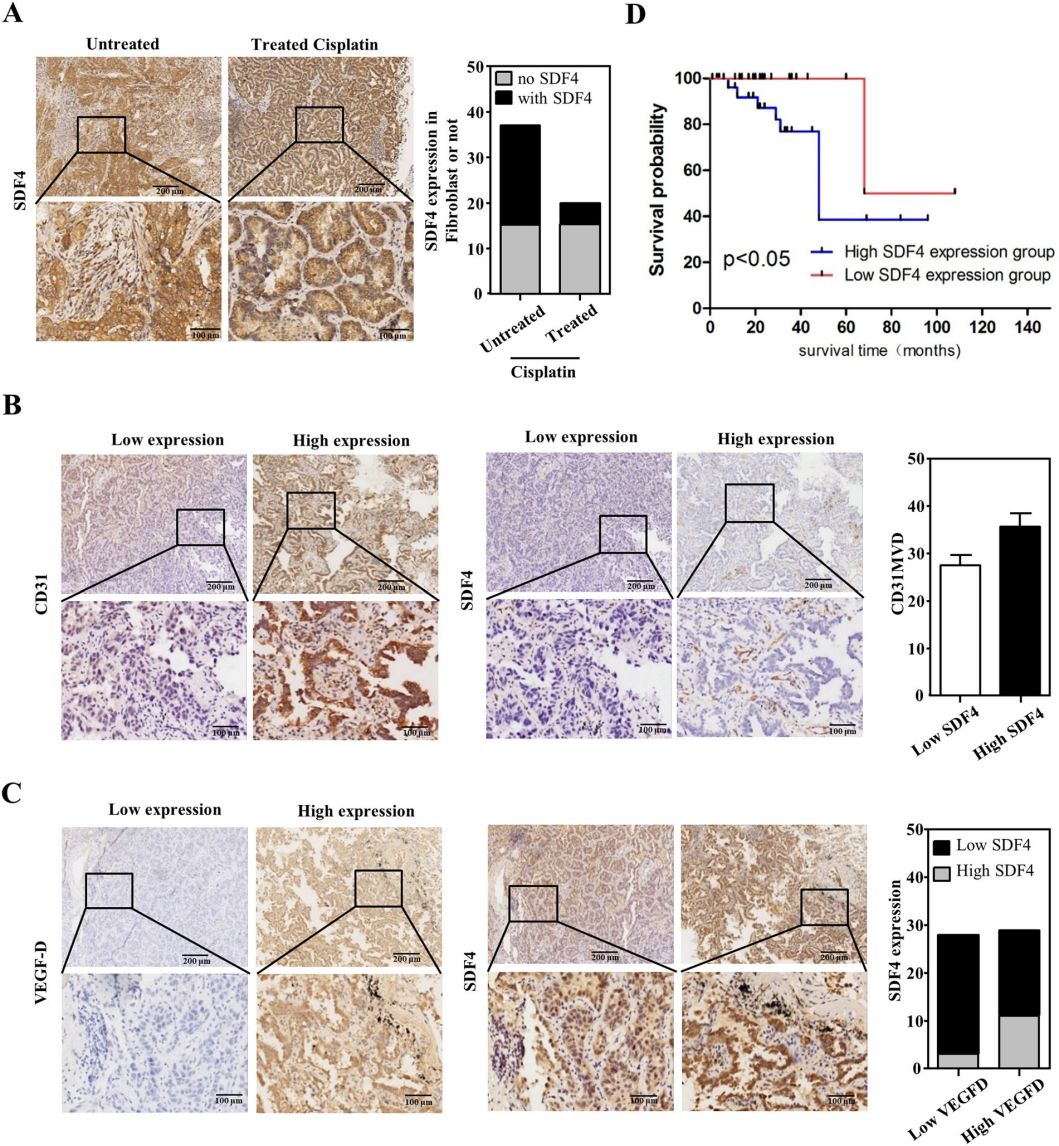
SDF4 is positively correlated with VEGF-D and the endothelial cell marker CD31 expression and poor survival of lung cancer patients treated with cisplatin. **A** Tissue specimens from 57 patients with lung cancer treated with or without cisplatin were stained for SDF4 expression. SDF4 was positively correlated with fibroblasts in cisplatin-treated lung cancer patients. **B**, **C** SDF4, VEGF-D, and CD31 staining was performed via IHC with their individual specific antibodies. SDF4 abundance was correlated with VEGF-D and CD31 signals in 57 lung cancer patients. **D** Correlation between SDF4 abundance and the survival rate of cisplatin-treated lung cancer patients. Differences between patient subsets in overall survival were determined via Kaplan–Meier plot analysis and log-rank tests. A two-tailed *p* < 0.05 was considered statistically significant. Statistical analysis was performed with SPSS v. 17.0 software (SPSS, USA).

As shown in Fig. 6A, specimens from 20 patients with lung cancer treated with cisplatin and 37 patients without cisplatin were collected to assess SDF4 abundance. Compared with 22 patients in the cisplatin-untreated group (*n* = 37), only 5 patients in the cisplatin-treatment subgroup (*n* = 20) showed lower SDF4 signals in fibroblasts (*p* = 0.013). We further explored the relationships between SDF4 expression and tumor angiogenesis in lung cancer specimens. According to IHC staining for the endothelial cell marker CD31 and VEGF-D (Fig. 6B, C), the microvascular density (MVD) was 27.46 ± 2.177 in the low SDF4 expression subgroup (*n* = 28) and 35.66 ± 2.835 in the high SDF4 expression subgroup (*n* = 29) (*p* = 0.026, Fig. 6B). Similarly, 11 specimens showed high VEGF-D abundance in the high SDF4 subgroup (11/29), whereas only 3 specimens showed high VEGF-D expression among the 28 samples in the low SDF4 expression subset (3/28) (*p* = 0.017, Fig. 6C). Importantly, Kaplan–Meier survival analysis confirmed that high SDF4 expression in fibroblasts was correlated with poor overall survival (Fig. 6D). Overall, high SDF4 expression was positively correlated with the endothelial cell marker CD31 and with VEGF-D signals in cisplatin-administered lung cancer patients.

## Discussion

Our results lend support to the emerging paradigm that maintains that stroma-derived signals contribute to tumor angiogenesis and suggests that targeting paracrine signaling mediated by chemo-treated CAFs could be a valid approach for improving therapeutic outcome in lung cancer. Because metastatic cancer is the major leading cause of cancer-related death and is currently incurable, it is critical that we target the microenvironment to prevent metastatic spread of cancer. Therefore, our data indicate that strategies can be developed to prevent chemotherapy-activated stroma-mediated cancer cell dissemination and subsequent metastasis. This prevention can be achieved by either discontinuing cisplatin in patients whose tumors show cisplatin-induced pro-metastatic changes or by combining cisplatin with agents that block stroma-mediated cancer cell dissemination, such as selective CXCR4 inhibitors, which would be useful not only in cisplatin treatment of localized lung cancer but also in treatment of metastatic lung cancer.

Platinum-doublet chemotherapy is routinely used for treatment of early-stage and metastatic NSCLC. Interestingly, it is increasingly recognized that chemotherapy can activate the tumor stroma to modulate tumor progression. Conventional chemotherapy is usually given at its maximum tolerated dose to maximize the effect on resistant cancer cells^42^. However, growing evidence has demonstrated that chemotherapeutic agents target both the tumor and the surrounding tumor stroma. In one study, treatment of prostate cancer, with Mitoxantrone and docetaxel, increased the expression of WNT16B in prostate fibroblasts, which enhanced cancer cell resistance to chemotherapy^14^. The combination of 5-flurouracil, oxaliplatin, and leucovorin results in additive or synergistic effects on colorectal cancer. These conventional chemotherapies activate CAFs to create a chemoresistant niche through IL-17A, which is a CSC maintenance factor that promotes self­renewal and tumor growth^19^. Particularly, doxorubicin can induce the expression of ELR motif-positive (ELR^+^) chemokines in breast fibroblasts, contributing to the expansion of stem-like tumor-initiating cells and promoting tumor aggression, and the addition of agents that target the tumor stroma can augment the efficacy of chemotherapeutic effects on tumor growth^43^. These findings agree with our observation that the tumor microenvironment contributes to cancer angiogenesis and increases the risk of tumor growth and metastasis during chemotherapy. In whole-genome transcriptomic analysis and a series of molecular and functional studies, the SDF4 chemokine–CXCR­4 paracrine signaling process stimulated by elevated p38 and ERK1/2 phosphorylation activity in endothelial cells was identified as an essential mechanism underlying the pro­angiogenesis and pro-metastatic activities of chemotherapeutic drug-­treated fibroblasts. Furthermore, and importantly, we demonstrated the clinical significance of this finding by showing that there is a significant induction of SDF4 in the stroma of CDDP-treated human cancer tissues. It is worth noting that the expression of previously reported CAF-secreted pro-angiogenic chemokines, such as CXCL12 (SDF­1α) and VEGF­A^44^, was not significantly induced by CDDP in fibroblasts. This outcome indicates that the chemotherapy­induced stroma alterations are different from nonchemo-regulated tumor progression.

Protein secretion is essential for intercellular communication during development and for the purpose of initiating various cellular processes, including differentiation and migration^45,46^. Cytokines in particular are one of the best-studied classes of secreted proteins, with broad effects on signaling molecules. Inadequate secretion of these cytokines results in several diseases, from chronic inflammation to cancer^47,48^. The synthesis of these cytokines and their subsequent transport to the cell surface must be tightly controlled. More importantly, organelles and molecular machineries that mediate the transport and secretion of these proteins must ensure regulated and directional transport and release of these cytokines. Interestingly, increasing evidence indicates that SDF4 (CAB45) plays a critical role in sorting secretory proteins. It binds calcium pumped into the TGN by the actin/cofilin/SPCA1 machinery via its EF-hand domains^49,50^. This binding in turn induces a conformational change and further triggers Cab45 binding of cargo molecules in the oligomeric state and sequesters them in certain TGN subdomains for subsequent export to the plasma membrane. Particularly, Cab45 recently has been reported to influence cancer progression and metastasis. For instance, the expression of Cab45 was elevated in human pancreatic cancer compared with human pancreatic duct epithelium^51^. Similar findings have been reported showing that secretion of Cab45 was abnormally increased upon treatment with the non-steroidal anti‐inflammatory drug Sulindac in colorectal cancer^52^. Moreover, the expression level of Cab45 influences the migrational capacity of cervical cancer, melanoma, and breast cancer cells. High expression levels of Cab45 promote expression of the EMT-related proteins N-cadherin, β-catenin, and vimentin^32^. Echoing these findings, our study showed that after chemotherapy treatment, lung cancer CAFs up­regulate SDF4 and further create a permissive environment for tumor cell angiogenesis and promotion of distant metastasis. Interestingly, in recent studies of zebrafish development, SDF4 was identified as an important component of vasculogenesis^53^. In addition, vasculogenesis and angiogenesis are the fundamental processes by which new blood vessels are formed. SDF4 proteins were present on endothelial cells and were upregulated under hypoxia. These findings showed an angiogenic role for SDF4 in zebrafish^53^. Indeed, Cab45 has a fundamental role in sorting of select cargoes in the Golgi, but we provide new clues to a novel mechanism for binding CXCR4, which was able to significantly enhance tumor angiogenesis ability and invasive behavior.

In summary, our findings suggest that systemic chemotherapy has a significant impact on the stroma that is associated with human lung cancers, including sustained activation of CAFs leading to pro­oncogenic and pro­angiogenic paracrine signaling activities. We delineated the signaling pathway that mediates this characteristic stromal response to chemotherapy and identified an effective way to attenuate it using CXCR4 inhibitors. Our results support the emerging paradigm that maintains that stroma­derived signals contribute to tumor pathology and suggests that targeting the paracrine signaling mediated by chemotherapeutic drug­treated CAFs could be a valid approach for improving therapeutic outcome in lung cancer.

## Materials and methods

### Cell culture and treatment

Human lung fibroblasts (HFL1) were obtained from the American Type Culture Collection (ATCC). HFL1 cells were cultured in Ham’s F-12 Kaighn’s modification medium (Gibco). MEFs were isolated from individual E13.5-E14.5 embryos generated by mating *Cebpd* null heterozygous mice. The MEFs used in this study were immortalized by E1A^54^. The mouse breast cancer 4T1 cells and immortalized *Cebpd*^+/+^ (7V7) and *Cebpd*^–/–^ (KO5) MEFs were maintained in Dulbecco’s modified Eagle’s medium. All culture media were supplemented with 10% fetal bovine serum (FBS), streptomycin (100 mg/ml), and penicillin (100 U/ml). HUVECs were purchased from the Bioresource Collection and Research Center of Taiwan and maintained in ECM (ScienCell) supplemented with 5% FBS, 1% endothelial cell growth supplement, and 1% penicillin/streptomycin. The recombinant protein, reagents, or inhibitors were then added individually for each experiment: 0.5-μg/ml SDF4 (Abnova), 30-μM CDDP (Sigma), or 10-μg/ml 5-FU (Sigma), 100-nM wortmannin, 10-μM PD98059, 10-μM SB203580, and 10-μg/ml AMD3100.

### Reverse-transcriptase polymerase chain reaction (RT-PCR)

Total RNA was isolated from experimental cells using TRIzol RNA extraction reagent. For RT-PCR analysis, total RNA was subjected to reverse transcription with SuperScript III (Invitrogen). The specific oligonucleotide primers used for the RT-PCR analysis are as follows: CEBPD, 5′-GCCATGTACGACGACGAGAG-3′ and 5′-TGTGATTGCTGTTGAAGAGGTC-3′; GAPDH, 5′-CCATCACCATCTTCCAGGAG-3′ and 5′-CCTGCTTCACCACCTTCTTG-3′; and α-SMA, 5′-CCCACCCAGAGTGGAGAA-3′ and 5′-ACATAGCTGGAGCAGCGTCT-3′; TGF-β, 5′-CACCGGAGAGCCCTGGATA-3′ and 5′-TGCCGCACACAGCAGTTC-3′; E-cadherin, 5′-TGGGCTGGACCGAGAGAGTT-3′ and 5′-ATCTCCAGCCAGTTGGCAGT-3′; N-cadherin, 5′-CACTGCTCAGGACCCAGAT-3′ and 5′-TAAGCCGAGTGATGGTCC-3′; Twist1, 5′-GTCCGCAGTCTTACGAGGAG-3′ and 5′-CCAGCTTGAGGGTCTGAATC-3′; Snail2, 5′-CTTTTTCTTGCCCTCACTGC-3′ and 5′-ACAGCAGCCAGATTCCTCAT-3′; SDF4, 5′-AGGCTCAACGAGGAACTCAA-3′ and 5′-ACCATGAACCTGAGCATTCC-3′; VEGF-A, 5′-CTTGCCTTGCTGCTCTACC-3′ and 5′-CACACAGGATGGCTTGAAG-3′; VEGF-B, 5′-AGCACCAAGTCCGGATG-3′ and 5′-GTCTGGCTTCACAGCACTG-3′; VEGF-C, 5′-TGCCGATGCATGTCTAAACT-3′ and 5′-TGAACAGGTCTCTTCATCCAGC-3′; VEGF-D, 5′-GTATGGACTCTCGCTCAGCAT-3′ and 5′-AGGCTCTCTTCATTGCAACAG-3′. SDF4, 5′-TTGACGACAACTGGGTGAAA-3′ and 5′-CGCACGAAGTGGCTATTTAAG-3′; SDF1, 5′-AGAGCCAACGTCAAGCATCT-3′ and 5′-ATCTGAAGGGCACAGTTTGG-3′.

### Conditioned medium collection

For the loss-of-function assay, conditioned medium was collected from HFL1 cells infected with lentiviruses bearing shβ-galactosidase (shC), shCEBPD (shD), or shSDF4 (shS) after 48 h. The experimental cells were further treated with or without CDDP or 5-FU for 24 h, which was replaced with 0.5% serum in F12K medium for another 24 h. Following centrifugation, the supernatants were collected and stocked for further assays in this study. For the gain-of-function assay, conditioned medium was collected from HFL1 cells 24 h after infection with lentiviruses bearing an empty vector (Ctl) or CEBPD expression vector (CD). Then, the cells were prepared as described above.

### Migration assays

HUVECs were seeded at 1 × 10^5^ per well in 24-well plates containing 8-μm pore inserts (BD Biosciences). Serum-free conditioned medium with or without different concentrations of SDF4 recombinant protein was placed in the upper wells after cells adhered to the inserts. DMEM with 10% FBS was added to the lower wells of the 24-well plates. The cells inside the insert were wiped away with cotton swabs after 16 h of incubation. The cells that had migrated to the bottom of the insert membrane were detected via DAPI staining. The total number of cells attached to the lower surface of the polycarbonate filter insert was determined at ×200 magnification under a fluorescence microscope.

### Reporter plasmids and luciferase assay

The *SDF4* reporter was constructed via PCR with the SDF4/−1462XhoI forward primer 5′-CCGCTCGAGCGGCCCGGCTCAGGCTCGCTGAG-3′ and SDF4/+155HindIII reverse primer 5′-CCCAAGCTTGGGGGGCCCCTCACTCACCGGTC-3′. The verified fragments were digested with XhoI and *Hind*III and then subcloned into a promoter PGL3-basic vector. To obtain different SDF4 fragments, we used PvuII/HindIII to obtain the 1.317-kb SDF4 promoter fragment, AatII/HindIII for the 1.159-kb fragment and AscII /HindIII for the 0.724-kb fragment. For the reporter assay, cells were transiently transfected using the SDF4 reporter and indicated expression vectors using TransIT-2020 transfection reagent (Mirus) according to the manufacturer’s instructions. Eighteen hours after transfection, the transfectants were treated with CDDP or 5-FU for another 6 h. The lysates of experimental cells were harvested to conduct the luciferase assay.

### ChIP assay

The ChIP assay was performed essentially as described by Wang et al.^55^. Briefly, following various treatments, including 30-μM CDDP or 10-μg/ml 5-FU, the experimental HFL1 cells were fixed with 1% formaldehyde. Cross-linked chromatin was then prepared and sonicated to an average size of 500 bp. The DNA fragments were immunoprecipitated with antibodies specific for CEBPD or control rabbit immunoglobulin G at 4 °C overnight. After reversal of the cross-linking, the immunoprecipitated chromatin was amplified by primers targeting specific regions of the gene’s genomic locus. The primers used detected sequences in the SDF4 genomic locus (5′-GACACGTCCT CGCTGTGCCAG-3′ and 5′-GCGACGCCTACGAAAACCTCAC-3′). The amplified DNA products were resolved via agarose gel electrophoresis and confirmed by sequencing.

### Cell viability assay

To assess the viability of cancer cells and stromal cells in response to anticancer drugs, MTT [3-(4,5-dimethylthiazol-2)-2,5-diphenyltetrazolium bromide] assays (Sigma) were conducted. HFL1 cells and A549 cells were treated with various concentrations of CDDP or 5-FU for 24 h. To assess the proliferation of HUVECs via the MTT assay, HUVECs were cultured in conditioned medium as described above or treated with different concentration of SDF4 recombinant protein for 24 and 48 h.

### Western blotting analysis

Cells were lysed in modified radioimmunoprecipitation assay buffer (modified RIPA) [50-mM Tris-HCl (pH 7.4), 150-mM NaCl, 1-mM EDTA, 1% NP-40, 0.25% sodium deoxycholate, 1-mM dithiothreitol, 1-mM phenylmethylsulfonyl fluoride, aprotinin (1 mg/ml), and leupeptin (1 mg/ml)]. Specific antibodies against α-tubulin (T6199, Sigma), CEBPD (sc-636, Santa Cruz Biotechnology), phospho-p44/42 (#4377, Cell Signaling), total p44/42 (#9102, Cell Signaling), phospho-p38 (#9211, Cell Signaling), total p38 MAPK (#9212, Cell Signaling), phospho-AKT (GTX61708, GeneTex), total AKT (GTX121937, GeneTex), SDF4 (10517-1-AP, Proteintech), and CXCR4 (60042-1- Ig, Proteintech) were used for western blotting.

### Short hairpin RNA (shRNA) assay

Lentiviruses were produced from Phoenix cells that had been cotransfected with various shRNA expression vectors in combination with pMD2.G and psPAX2. After determination of the viral infection efficiency, HFL1 cells were infected for 48 h with shC, shD, or shS lentiviruses, and HUVECs were infected for 48 h with shC and shCXCR4 lentiviruses, each at a multiplicity of infection of 10. The shRNA oligo sequences used in the lentiviral expression vectors were as follows: shC, 5′-CCGGTGTTCGCATTATCCGAACCATCTCGAGATGGTTCGGATAATGCGAACATTTTTG-3′; shD, 5′-CCGGGCTGTCGGCTGAGAACGAGAACTCGAGTTCTCGTTCTCAGCCGACAGCTTTTT-3′; shP, 5′- CCGGGAGGAGCTCAGTATGTTTCATCTCGAGATGAAACATACTGAGCTCCTCTTTTTTG-3′, shS, 5′-CCGGCCGGAGGAAGCTGATGGTCATCTCGAGATGACCATCAGCTTCCTCCGGTTTTTG-3′, and shCXCR4, 5′-CCGGGCGTGTAGTGAATCACGTAAACTCGAGTTTACGTGATTCACTACACGCTTTTTG-3′. The lentiviral knockdown expression vectors were purchased from the National RNAi Core Facility located at the Genomic Research Center of the Institute of Molecular Biology, Academia Sinica, Taiwan.

### Immunofluorescence assays

Tissue sections were cut from frozen tumor blocks at a thickness of 5 μm and placed onto precoated slides. The slides were treated with protein blocker (Biovision) for 1 h. For antigen retrieval, the slides were heated to 121 °C in 10-mM citrate buffer (pH 6) for 5 min. The slides were then washed with phosphate-buffered saline and incubated with specific antibodies recognizing CEBPD (sc-636; Santa Cruz), α-SMA (ab119952; Abcam), and CD31 (77699S; Cell signaling) at a dilution of 1:350 for 1 h. The slides were then incubated with Alexa488-, 555-, or 594-conjugated secondary antibodies for 1 h at room temperature at a 1:200 dilution. Images were acquired with a laser scanning confocal microscope (TE2000EPFS-C1-Si, Nikon).

HFL1 cells were grown on coverslips treated with or without CDDP or 5-FU for 24 h. Similarly, HUVECs were grown on coverslips treated with or without SDF4 for 24 h. The cells were fixed in ice-cold ethanol for 20 min and incubated with antibody against α-SMA (1:100 dilution; ab119952, Abcam), SDF4 (1:100 dilution, 10517-1-AP, Proteintech), and CXCR4 (1:100 dilution, 60042-1- Ig, Proteintech). For immunofluorescence analysis, samples were incubated with Alexa488- or 568-conjugated secondary antibodies (Invitrogen) at 1:200 dilution. The samples were washed with 0.1% Tween 20 in PBS and mounted on coverslips, and images were acquired with a laser scanning confocal system consisting of a BX51 microscope (Olympus) with a DP70 digital camera system and DP Controller software (Olympus).

### Co-immunoprecipitation assay

The lysates of HUVECs were processed for membrane protein extraction using a Mem-PER Mammalian Membrane Protein Extraction Reagent Kit (Pierce) following the manufacturer’s protocol. The membrane proteins were collected and incubated with or without SDF4 recombinant protein and co-incubated with specific antibodies recognizing SDF4 (1:100 dilution, 10517-1-AP, Proteintech), CXCR4 (1:100 dilution, 60042-1-Ig, Proteintech), and GST (1:100 dilution, sc-138, Santa Cruz) at 4 °C for at least 4 h. Protein-A/G agarose beads were added to the lysates, and the mixtures were incubated and rotated at 4 °C for 1 h. The beads were collected using centrifugation and washed three times with the modified RIPA buffer. The proteins bound to the beads were eluted by adding electrophoresis sample buffer and then subjected to western blot analysis.

### Xenograft animal study

To construct A549 xenografts, 2 × 10^6^ A549 cells mixed with 1 × 10^6^ shC-HFL1, 1 × 10^6^ shD-HFL1, or 1 × 10^6^ shD-HFL1 were inoculated subcutaneously into the dorsal rear flanks of NOD-SCID mice. Similarly, 2 × 10^6^ A549 cells mixed with 1 × 10^6^ shC-HFL1, 1 × 10^6^ shS- HFL1, or 1 × 10^6^ shS-HFL1 were inoculated subcutaneously into the dorsal rear flanks of NOD-SCID mice. For in vivo experiments of mice treated with or without CDDP, beginning 1 week after inoculation with tumor cells, the mice were treated weekly with an intraperitoneal injection of CDDP (5 mg/kg) dissolved in 1% (w/v) DMSO or with DMSO only. Tumor size was measured with external calipers, and tumor volume was calculated using the standard formula: *V* = (*w* × *l*^2^) × 0.52, where *l* is the length and *w* is the width of the tumor. The animals were sacrificed 12 weeks after inoculation with tumor cells. The spleen, kidneys, lungs, and liver of mice were harvested for analysis with an IVIS Spectrum Imaging System 200 (Caliper) to determine metastatic activity. All experiments on mice were performed according to the guidelines of our institute (the Guide for Care and Use of Laboratory Animals, National Cheng Kung University). The animal use protocol described below was reviewed and approved by the Institutional Animal Care and Use Committee (IACUC).

### Allograft animal study

Six-week-old female C57BL/6 mice were purchased from the National Laboratory Animal Center. Cebpd-deficient mice (on a C57BL/6 background) were a gift from E. Sterneck (National Cancer Institute, Frederick, MD). Stably transfected LLC1 cell lines expressing m-cherry were inoculated subcutaneously into the dorsal rear flanks of C57BL/6 mice. In experiments of mice treated with or without CDDP, beginning 1 week after inoculation with tumor cells, mice were injected intraperitoneally every week with CDDP (5 mg/kg) dissolved in 1% (w/v) DMSO or with DMSO only. Tumor size was measured by external calipers, and tumor volumes were calculated with the standard formula: *V* = (*w* × *l*^2^) × 0.52, where *l* is length and *w* is width of the tumor. In orthotopic model, the luciferase expressing LLC1 cells (LLCI-Luc2) were orthotopically inoculated into the left lungs of C57BL/6 mice or *Cebpd*-deficient mice. In experiments of mice treated with or without CDDP (5 mg/kg) and/or AMD3100 (1.25 mg/kg) beginning 1 week after inoculation with tumor cells, mice were injected intraperitoneally every week. Animals were sacrificed 6 weeks after inoculation with tumor cells. The spleen, kidneys, lungs, and liver of mice were harvested for analysis with an IVIS Spectrum Imaging System 200 (Caliper) to determine metastatic activity. All experiments on mice were performed according to the guidelines of our institute (the Guide for Care and Use of Laboratory Animals, National Cheng Kung University). The animal use protocol described below has been reviewed and approved by the IACUC.

### Tube formation assays

Matrigel was precoated onto 48-well plates and allowed to solidify for 1 h at 37 °C. HUVECs (3 × 10^4^) were cultured in ECM, treated with various concentrations of SDF4 recombinant protein, or combined with conditioned medium from HFL1 cells. After 6 h of incubation, the experimental cells were fixed with 4% paraformaldehyde, and a blinded observer assessed the morphology of the tubes. Randomly chosen fields were photographed at ×100 magnification, and the tube-like structures were quantified by counting the number of intersections between branches of endothelial cell networks.

### Mouse Matrigel plug assay

Male C57BL/6 mice (6–8 weeks old) were used in the Matrigel plug assay. *Cebpd*^−/−^ mice (on a C57BL/6 background) were a gift from Dr E. Sterneck^56^. Mice were injected with 450 μl of Matrigel (BD Biosciences) supplemented with 800-ng/ml bFGF (Peprotech), 150-ng/ml VEGF-165 (GFH44; Cell guidance systems), or 500-ng/ml SDF4 and 100-μg/ml heparin sulfate. Five days later, mice were killed, and the Matrigel plugs were removed. To quantitate the formation of functional blood vessels, hemoglobin levels were measured using a Drakin’s reagent kit (D5941; Sigma).

### Patients

In total, 57 locally advanced lung cancer patients (Stages III and IV) treated at the Sixth Affiliated Hospital of Sun Yat-sen University were enrolled in the present study. Among them, 20 patients had received at least one cycle of cisplatin-based chemotherapy, and 37 patients had been treated with non-cisplatin chemotherapy. All of the patients were followed up at 3-month intervals. Overall survival was defined as the time from diagnosis to the date of death or when censored at the latest date if patients were still alive. This study was approved by the Clinical Ethics Review Board at Cancer Center of Sun Yat-sen University (Guangzhou, China).

### IHC staining

Immunohistochemical staining was performed as previously reported^57^. The primary antibodies used in this study were mouse anti-CD31 monoclonal antibody (1:100 dilution; Zhongshan JinQiao, ZM-0044, China), rabbit anti-SDF4 polyclonal antibody (1:80 dilution; Proteintech, 10517-1-AP, USA), and mouse anti-VEGF-D monoclonal antibody (1:50 dilution; R&D systems, MAB286, USA). In addition, a negative control was employed by replacing the specific primary antibody with nonimmune serum immunoglobulins at 1:200 dilution. Brown granules in the nucleus or cytoplasm were considered to indicate positive staining. The expression level of SDF4 and VEGF-D was evaluated by assessing staining intensity and extent. We scored the staining intensity as follows: negative and bordering (score 0); weak (score 1); moderate (score 2); or strong (score 3). Staining extent was divided into five grades according to the percentage of elevated staining stained cells in the field: negative (score 0), 0–25% (score 1), 26–50% (score 2), 51–75% (score 3), and 76–100% (score 4). The staining intensity score was multiplied by the staining extent score to obtain the overall SDF4 and VEGF-D expression score. MVD was determined by counting the CD31-positive blood vessels in five random 100X fields. Two independent pathologists (X.-J.F. and H.-L.L.), blind to follow-up data, were involved in IHC staining scoring. A third pathologist would arbitrate when any discrepancy arose between the two pathologists.

### Statistical analysis

Differences between patient subsets in overall survival were determined via Kaplan–Meier plot analysis and log-rank tests. A two-tailed *p* < 0.05 was considered statistically significant. Statistical analysis was performed with SPSS v. 17.0 software (SPSS, USA).

## Supplementary information

Supplementary figure and legends

Supplementary figure 1

Supplementary figure 2

Supplementary figure 3

Supplementary figure 4
