# Supplementary material for: Fibroblast CEBPD/SDF4 axis in response to chemotherapy-induced angiogenesis through CXCR4
Source: Cell Death Discov. 2021 May 6;7:94. doi: 10.1038/s41420-021-00478-0 (PMC8099881; doi:10.1038/s41420-021-00478-0)
Supplement: Supplementary file 1 — Supplementary figure and legends [file 41420_2021_478_MOESM1_ESM.docx]

**Supplementary Figures legends**

**Figure S1. CEBPD contributes to myofibroblast differentiation. (A)** CEBPD is responsive to CDDP and 5-FU treatment in HFL1 cells. **(B)** Exogenous CEBPD expression activates *N-cadherin,* *snail*, *slug* and *TWIST* transcription in HFL1 cells. HFL1 cells were infected with lentivirus bearing pAS3W-control (Ctl) or pAS3W-CEBPD expression vectors (CD). *E-cadherin*, *N-cadherin*, *snail2* and *TWIST1* mRNA expression was determined by an RT-PCR assay. **(C)** The migration of HFL1 cells was assessed using a Boyden chamber assay. HFL1 cells were infected with lentivirus bearing Ctl or CD expression vectors. **(D)** LLC1-Luc2 cells were orthotopically inoculated into the lung of C57BL/6 mice or *Cebpd*-deficient mice. The experimental mice were treated with or without CDDP as indication after inoculation with tumor cells. Representative in vivo bioluminescent images and total tumor flux of LLC1-Luc2-bearing mice in each group shown at 5^th^ week. n=8 per group.

**Figure S2. Gene expression changes in *Cebpd*-deficient lung fibroblasts in response to CDDP treatment. (A)** Analysis of gene expression changes in HFL1 cells via transcript microarray quantification. **(B)** The relative mRNA levels in HFL1 cells infected with shβ-galactosidase (shC) lentiviruses compared with cells infected with shCEBPD (shD) lentiviruses upon CDDP treatment.

**Figure S3. Biological processes enriched by SDF4-regulated genes and functions. (A) Enrichment analysis using pathway maps in the angiogenesis folder in MetaCore. Next-generation sequencing (NGS)** analysis was performed using total RNA harvested from HUVECs treated with or without 0.5 μg/ml SDF4 for 6 h. Genes (1881) responsive to SDF4 were extracted for MetaCore analysis. **(B)** An MTT assay was conducted to assess the proliferation of HUVECs in response to SDF4 at concentrations of 0.25 μg/ml, 0.5 μg/ml or 1 μg/ml for 24 or 48 h.

**Figure S4. The ERK1/2 and p38 MAPK pathway contributes to SDF4-induced VEGFD expression in endothelial cells. (A)** Morphological images of *in vitro* tube formation of HUVECs following treatment with SDF4 (0.5 μg/ml) with/without AMD3100 (10 μg/ml and 20 μg/ml), **(B)** wortmannin (10 nM and 100 nM), PD98059 (5 μM and 10 μM) or SB203580 (5 μM and 10 μM). **(C)** The effect on *VEGF* transcription in SDF4-treated HUVECs. An RT-PCR assay was conducted using total RNA from SDF4-treated HUVECs over the indicated time courses.
