# Supplementary figures and images for: Fibroblast CEBPD/SDF4 axis in response to chemotherapy-induced angiogenesis through CXCR4
Source: Cell Death Discov. 2021 May 6;7:94. doi: 10.1038/s41420-021-00478-0 (PMC8099881; doi:10.1038/s41420-021-00478-0)

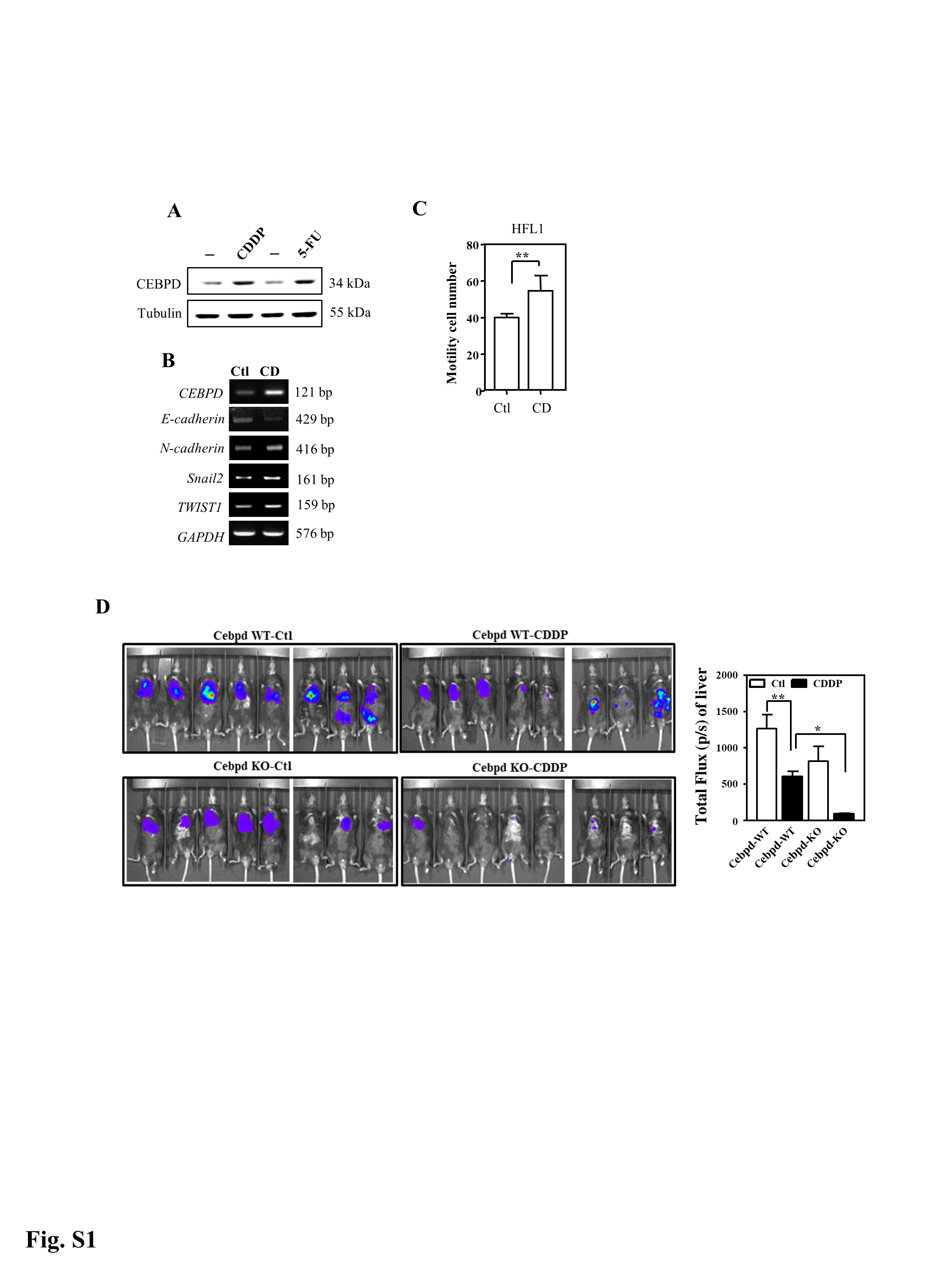

Supplement: Supplementary file 2 — Supplementary figure 1 [file 41420_2021_478_MOESM2_ESM.tif]

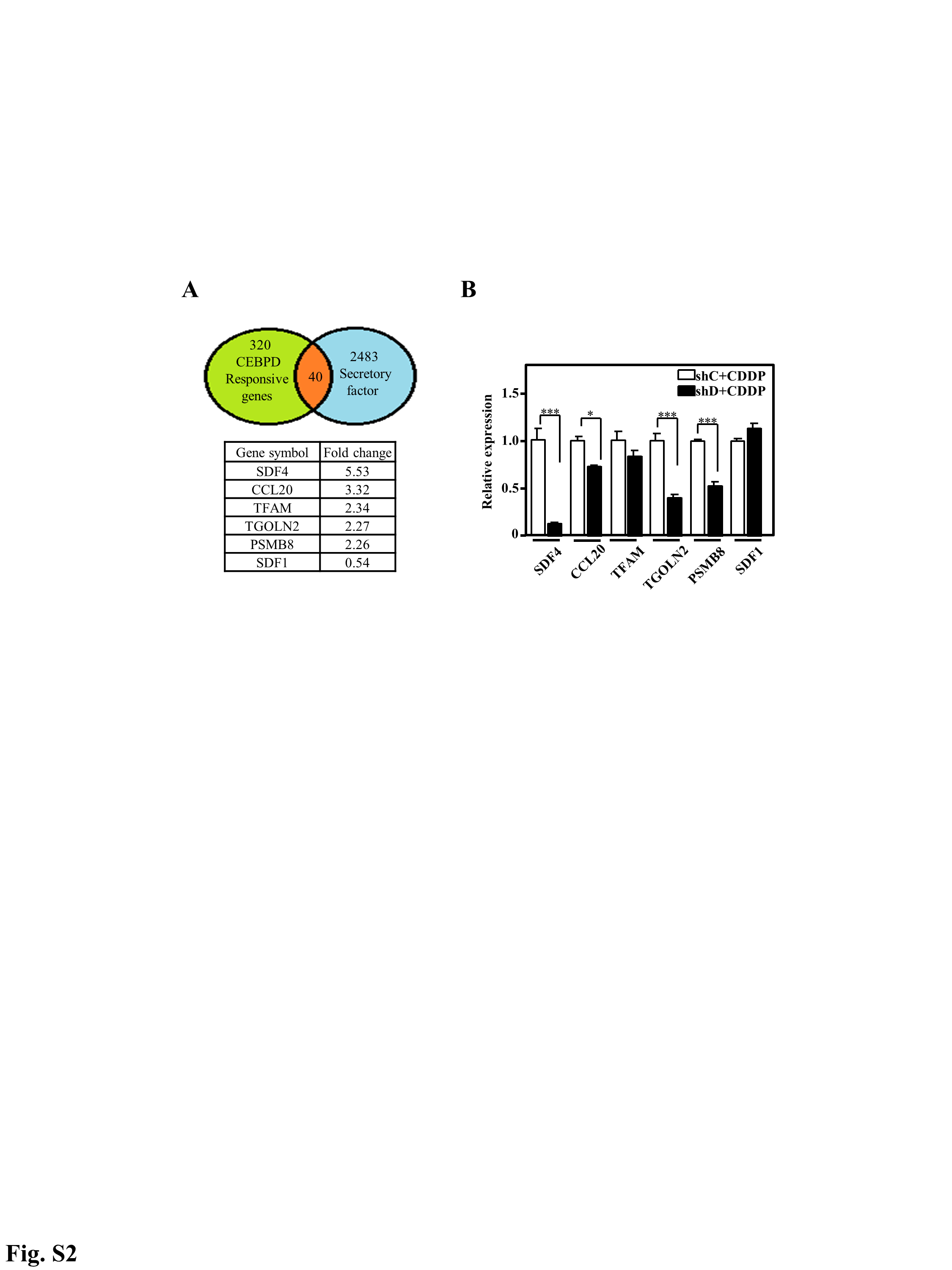

Supplement: Supplementary file 3 — Supplementary figure 2 [file 41420_2021_478_MOESM3_ESM.tif]

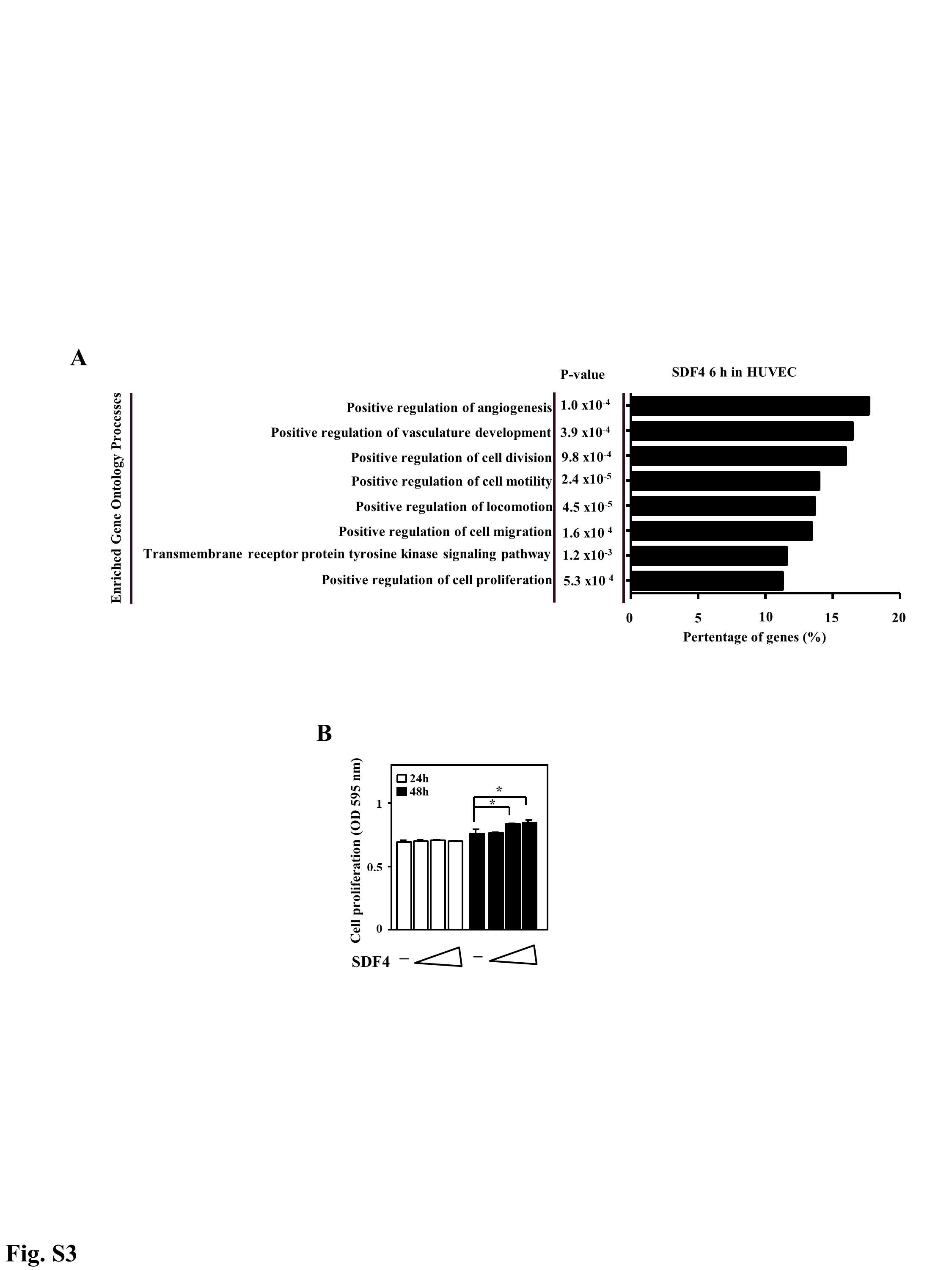

Supplement: Supplementary file 4 — Supplementary figure 3 [file 41420_2021_478_MOESM4_ESM.tif]

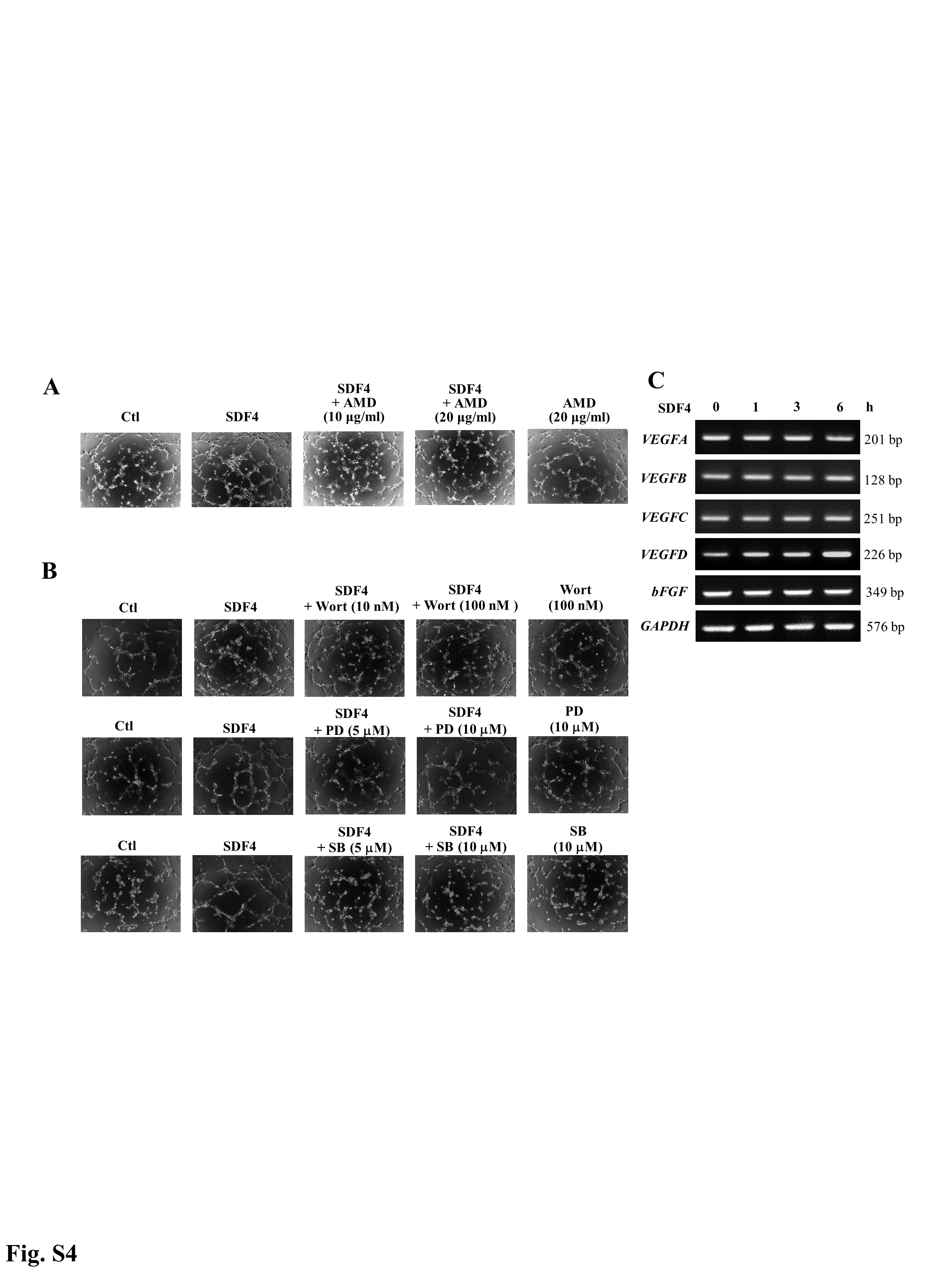

Supplement: Supplementary file 5 — Supplementary figure 4 [file 41420_2021_478_MOESM5_ESM.tif]
